# Associations between polysubstance use and psychiatric comorbidities

**DOI:** 10.1192/j.eurpsy.2023.1608

**Published:** 2023-07-19

**Authors:** R. Fernández Fernández, P. del Sol Calderón, Á. Izquierdo de la Puente, M. Vizcaíno da Silva

**Affiliations:** 1 Psychiatry, Hospital Universitario Infanta Cristina, Parla; 2 Psychiatry, Hospital Puerta de Hierro, Majadahonda; 3 Psychiatry, Hospital de El Escorial, San Lorenzo de El Escorial, Spain

## Abstract

**Introduction:**

Polydrug use studies mention demographic and socioeconomic factors that may influence this problem. One of them is the existence of psychiatric comorbidity; Rentrop’s study (Rentrop et al., 2014) finds in a sample of 50 patients that all patients had at least one axis I disorder, 90% at least one axis II disorder, which may compromise the outcome of detoxification and dehabituation treatments (Rentrop et al., 2014). Another study found that 44.9% of patients admitted to a psychiatric unit are polydrug users (Karam et al., 2002).

**Objectives:**

To study the possible association of polydrug use with psychiatric comorbidity in patients admitted to a general hospital and presenting drug use.

**Methods:**

We made a descriptive retrospective study through the use of electronic medical records. The drug use history was obtained for all patients admitted to the inpatient service of a general hospital during a 3-year period.

**Results:**

More cases of poly-consumption together with psychiatric comorbidity are found than expected in the χ² Test, with significant results (χ² = 27.2; p<0.001). The mean age of the patient with poly-consumption and psychiatric comorbidity is 34.9 years.

**Table:** 

**Psychiatric comorbidity**	**Polydrug use**	No	Yes	Total
No	Observed	296	0	296
	Expected	284	11.64	296
Yes	Observed	217	21	238
	Expected	229	9.36	238
Total	Observed	513	21	534
		513	21	534

**Conclusions:**

Psychiatric comorbidity in patients with polydrug use may be overlooked (Kruckow et al. 2016). Identifying patients with dual diagnosis is important given that these patients suffer decreased treatment compliance and life expectancy compared with single-diagnosis patients (Kruckow et al., 2016).

**References:**

Rentrop, M., Zilker, T., Lederle, A., Birkhofer, A., & Hörz, S. (2014). Psychiatric comorbidity and personality structure in patients with polyvalent addiction. Psychopathology, 47(2), 133–140. https://doi.org/10.1159/000351784

Kruckow, L., Linnet, K., & Banner, J. (2016). Psychiatric disorders are overlooked in patients with drug abuse. Danish medical journal, 63(3), A5207.

**Disclosure of Interest:**

None Declared

